# Visualization of Oxytocin Release that Mediates Paired Pulse Facilitation in Hypothalamic Pathways to Brainstem Autonomic Neurons

**DOI:** 10.1371/journal.pone.0112138

**Published:** 2014-11-07

**Authors:** Ramón A. Piñol, Heather Jameson, Anastas Popratiloff, Norman H. Lee, David Mendelowitz

**Affiliations:** 1 Department of Pharmacology and Physiology, The George Washington University, Washington, DC, United States of America; 2 Institute for Biomedical Sciences, The George Washington University, Washington, DC, United States of America; 3 Center for Microscopy and Image Analysis, The George Washington University, Washington, DC, United States of America; University of Pecs Medical School, Hungary

## Abstract

Recent work has shown that oxytocin is involved in more than lactation and uterine contraction. The paraventricular nucleus of the hypothalamus (PVN) contains neuroendocrine neurons that control the release of hormones, including vasopressin and oxytocin. Other populations of PVN neurons do not release hormones, but rather project to and release neurotransmitters onto other neurons in the CNS involved in fluid retention, thermoregulation, sexual behavior and responses to stress. Activation of oxytocin receptors can be cardioprotective and reduces the adverse cardiovascular consequences of anxiety and stress, yet how oxytocin can affect heart rate and cardiac function is unknown. While anatomical work has shown the presence of peptides, including oxytocin, in the projections from the PVN to parasympathetic nuclei, electrophysiological studies to date have only demonstrated release of glutamate and activation of fast ligand gated receptors in these pathways. In this study, using rats, we directly show, using sniffer CHO cells that express oxytocin receptors and the Ca^2+^ indicator R-GECO, that optogenetic activation of channelrhodopsin-2 (ChR2) expressing PVN fibers in the brainstem activates oxytocin receptors in the dorsomotor nucleus of the vagus (DMNV). We also demonstrate that while a single photoactivation of PVN terminals only activates glutamatergic receptors in brainstem cardiac vagal neurons (CVNs), neurons that dominate the neural control of heart rate, both the paired pulse facilitation, and sustained enhancement of glutamate release in this pathway is mediated by activation of oxytocin receptors. Our results provide direct evidence that a pathway from the PVN likely releases oxytocin and enhances short-term plasticity of this critical autonomic connection.

## Introduction

Recent work has shown that vasopressin neurons in the paraventricular nucleus of the hypothalamus (PVN) are critical for the cardiovascular responses to challenges such as stress and dehydration, and are involved in the maintenance and/or generation of cardiovascular diseases, including hypertension [1], [2]–[5]. However the PVN is a highly heterogeneous nucleus. Whereas vasopressin (AVP) neurons in the PVN are sympathoexcitatory, and activation of vasopressin receptors inhibits cardioinhibitory parasympathetic cardiac vagal neurons (CVNs) [6], recent work has shown activation of oxytocin receptors can be cardioprotective and reduces the adverse cardiovascular consequences of anxiety and stress [7], [8], [9].

Yet how oxytocin can affect heart rate and cardiac function is unknown. CVNs generate parasympathetic activity to the heart and are responsible for maintaining a normal heart rate by suppressing the cardioacceleratory influences of sympathetic activity and the high intrinsic firing rate of cardiac pacemaker cells in the sino-atrial node [10]. While anatomical work has shown the presence of peptides, including oxytocin, in the projections from the PVN to parasympathetic nuclei [11], electrophysiological studies to date have only demonstrated release of glutamate and activation of fast ligand gated receptors in these pathways [12], [13]. In this study we test if photoactivation of channelrhodopsin-2 (ChR2) expressing PVN fibers in the brainstem releases oxytocin and activates oxytocin receptors using sniffer CHO cells that are engineered to be highly sensitive to oxytocin by co-expression of oxytocin receptors and the Ca^2+^ indicator R-GECO. We also test the hypothesis that stimulation of the pathway from the PVN to CVNs activates oxytocin receptors and elicits functional changes in synaptic plasticity within this excitatory cardioprotective pathway.

## Materials and Methods

### Ethical approval

All efforts were made to minimize the number of animals used and to avoid any possible discomfort. All animal procedures were performed in compliance with the institutional guidelines at The George Washington University (Washington DC, USA) and are in accordance with the recommendations of the Panel on Euthanasia of the American Veterinary Medical Association and the National Institutes of Health publication Guide for the Care and Use of Laboratory Animals. The GWU Institutional Animal Care and Use Committee (IACUC) specifically approved this study.

### Lentiviral vector production

Lentiviral plasmids pLenti-Syn-hChR2(H134R)-EYFP-WPRE, packaging plasmid pCMV-ΔR8.74 and envelope plasmid pMD2.G were all kindly donated by K. Deisseroth (Stanford University, Stanford, CA, USA). Lentiviral particles with VSVg pseudotype were produced according to customary protocols as described before [12]. All used batches of virus had a titer between 2×10^8^ and 2×10^9^ transducing units (TU) per ml.

### Stereotactic injections, cardiac labelling and immunohistochemistry

Neonatal (P5, 5–8 gms) Sprague Dawley rats, of either sex, were anesthetized by hypothermia and mounted in a stereotactic apparatus with a neonatal adapter (Stoelting, Wood Dale, IL, USA). The viral vector (50–75 nl) was injected into the PVN, after which the pipette was left in place for 10 minutes, then the incision was closed and the animal was allowed to recover. Animals that had injections outside the boundaries of the PVN were excluded from further analysis.To label CVNs for electrophysiology, a right thoracotomy was performed and 20 µl of X-rhodamine-5-(and 6)-isothiocyanate (XRITC; Invitrogen, Eugene, OR) was injected into the pericardial sac at the base of the heart, as described previously [14], [15].

To examine the co-localization of ChR2-EYFP and oxytocin in PVN fibers within the dorsal motor nucleus of the vagus (DMNV) slices (200 microns thick) were soaked in 10% formalin for one hour and were processed for oxytocin and EYFP immunohistochemistry using the following primary antibodies (overnight incubation at 22–24°C): rabbit anti-oxytocin antibody (1∶15000 dilution; T-4084, Bachem, Torrance, CA) and mouse anti-GFP/EYFP (1∶500 dilution; ab38689, Abcam, Cambridge, MA). As secondary antibodies we used goat anti rabbit Alexa Fluor 405 and chicken anti-mouse Alexa Fluor 488 (all 1∶200 dilution and 4 h incubation at 22–24°C; Life Technologies, Carlsbad, CA).

### Sniffer CHO cells and Ca^2+^ imaging

CHO cells were transfected with pcDNA3.1+ containing human OXTr cloned in at EcoRI (5′) and XhoI (3′) (plasmid obtained from Missouri S&T cDNA Resource Center; www.cdna.org) using lipofectamine and stable over-expression was achieved by genetcin (500 µg/ml) selection. OXTr-expressing CHO-cells were then plated and transiently transfected to also express the red fluorescent genetically encoded Ca^2+^ indicator (R-GECO; plasmid kindly donated by Robert Campbell, University of Alberta, Canada; Addgene plasmid 32444) [16] with Fugene 6. To examine the selectivity, affinity and responsiveness of these OXTr-expressing CHO cells we examined the dose-response relationship of these sniffer cells to both oxytocin and vasopressin. To study the activation of oxytocin receptors upon stimulation of PVN fibers in the DMNV sniffer cells were pipetted onto the dorsal vagal complex of brain stem slices of animals previously injected with Chr2-EYFP-expressing lentivirus in the PVN. Only sniffer CHO cells within boundaries of the dorsal motor nucleus of the vagus (DMNV) were analyzed and these cells were 7.5±0.5 microns distant from the closest ChR2 containing PVN fiber. Imaging was performed on a confocal microscope system consisting of an upright Zeiss Axio Examiner Z1 microscope, with a W Plan Apocromat 20x/1.0 objective, equipped with Carl Zeiss 710 confocal hardware. Z-series spectral image sets were used to produce two channel image sets representing ChR2-EYFP fibers and sniffer cells, by applying off-line a linear spectral un-mixing protocol. For Ca^2+^ imaging upon photo-excitation of the ChR2 fibers, images measured 128×128 pixels taken at 2.3 zoom factor and bi-directional scanning. Thus the pixel measured 1.44 µm, providing sufficient cellular and temporal resolution. Images were obtained every 76 msec.

### Slice preparation, electrophysiology and drugs

Rats (5–7 weeks old and 150–250 gms) were anesthetized with isoflurane, sacrificed, and transcardially perfused with ice-cold glycerol-based aCSF (252 mM glycerol, 1.6 mM KCl, 1.2 mM NaH_2_PO_4_, 1.2 mM MgCl_2_, 1.2 mM CaCl_2_, 18 mM NaHCO_3_, 11 mM glucose, perfused with 95% O_2_ and 5% CO_2_, pH = 7.4). The brain was carefully removed and brainstem slices (300 µm) were obtained using a compresstome (VF- 300; Precisionary Instruments Inc. Greenville, NC, USA) (all in the glycerol-based aCSF), and forebrain slices containing the PVN were cut (150 µm for injection site verification). Slices were allowed to recover at 32°C for 15 minutes in NMDG-based aCSF (110 mM NMDG, 2.5 mM KCl, 1.2 mM NaH_2_PO_4_, 25 mM NaHCO_3_, 25 mM glucose, 0.5 mM CaCl_2_ and 10 mM MgSO_4_; pH titrated with concentrated HCl to 7.3–7.4, perfused with 95% O_2_ and 5% CO_2_,), before being transferred to 22–24°C aCSF (125 mM NaCl, 3 mM KCl, 2 mM CaCl2, 26 mM NaHCO_3_, 5 mM glucose, and 5 mM HEPES, perfused with 95% O_2_ and 5% CO_2_, pH = 7.4) in which experiments were performed. Identified CVNs in DMNV were imaged with differential interference contrast optics, infrared illumination, and infrared-sensitive video detection cameras to gain better spatial resolution. A *473 nm* blue *CrystaLaser (Reno, NV, USA), attached to the microscope was used for selective photostimulation of ChR2*. Patch pipettes (2.5–4.5 MOhm) contained 135 mM K gluconic acid, 10 mM HEPES, 10 mM EGTA, 1 mM CaCl_2_, 1 mM MgCl_2_ and 2 mM Na-ATP, pH = 7.3. Synaptic activity from identified CVNs was recorded at −80 mV. Voltage-clamp whole-cell recordings were made with an Axopatch 200B and pClamp 9 software (Axon Instruments, Union City, CA, USA). Control recordings were performed in the presences of strychnine (1 µM) and gabazine (25 µM). d-2-amino-5-phosphonovalerate (AP-5; 50 µM), 6-cyano-7-nitroquinoxaline-2,3-dione (CNQX; 50 µM), (d(CH_2_)_5_^1^,Tyr(Me)^2^,Thr^4^,Orn^8^,des-Gly-NH_2_^9^)-Vasotocin (OTA; 10 µM; Bachem BioSciences, Torrance CA, USA) and (Thr^4^,Gly^7^)-Oxytocin (TGOT; 0.5 µM; Bachem BioSciences, Torrance CA, USA) were added to the perfusate as indicated. Non-selective stimulation of synapses surrounding CVNs was performed by placing a stimulus isolator (A.M.P.I., Jerusalem, Israel) 200–400 µm lateral to the recorded CVN. Electrical stimuli of 1-ms duration were used stimulus at an intensity that was 1.5 times of the minimum intensity that evoked a response in CVNs.

### Analysis and statistics

Synaptic events and peak amplitudes were analyzed using Clampfit 10.1 (Axon Instruments) and MiniAnalysis (Synaptosoft version 4.3.1) software. All data are represented by mean ± SEM. For analysis of paired-pulse response (PPR) and five times 3 ms stimulation, we excluded failures from analysis as there was no difference between failure rates in control and OTA conditions. EPSC frequency in the prolonged bursting stimulation experiments were normalized to the frequency of EPSCs during the first 5 s of each experiment. The Ca^2+^ responses were binned in 0.5 s periods in the graph and in 1 s periods for statistical analysis. Results were tested for statistical significance using Student's paired *t* tests. For examining the OTA effect on five pulses stimulation we used a repeated measures two-way analysis ANOVA, and for Ca^2+^ imaging and time course of the facilitation following bursting stimulations one-way ANOVA with repeated measures analysis (GraphPad Prism 5 software).

## Results

**Figure 1:**
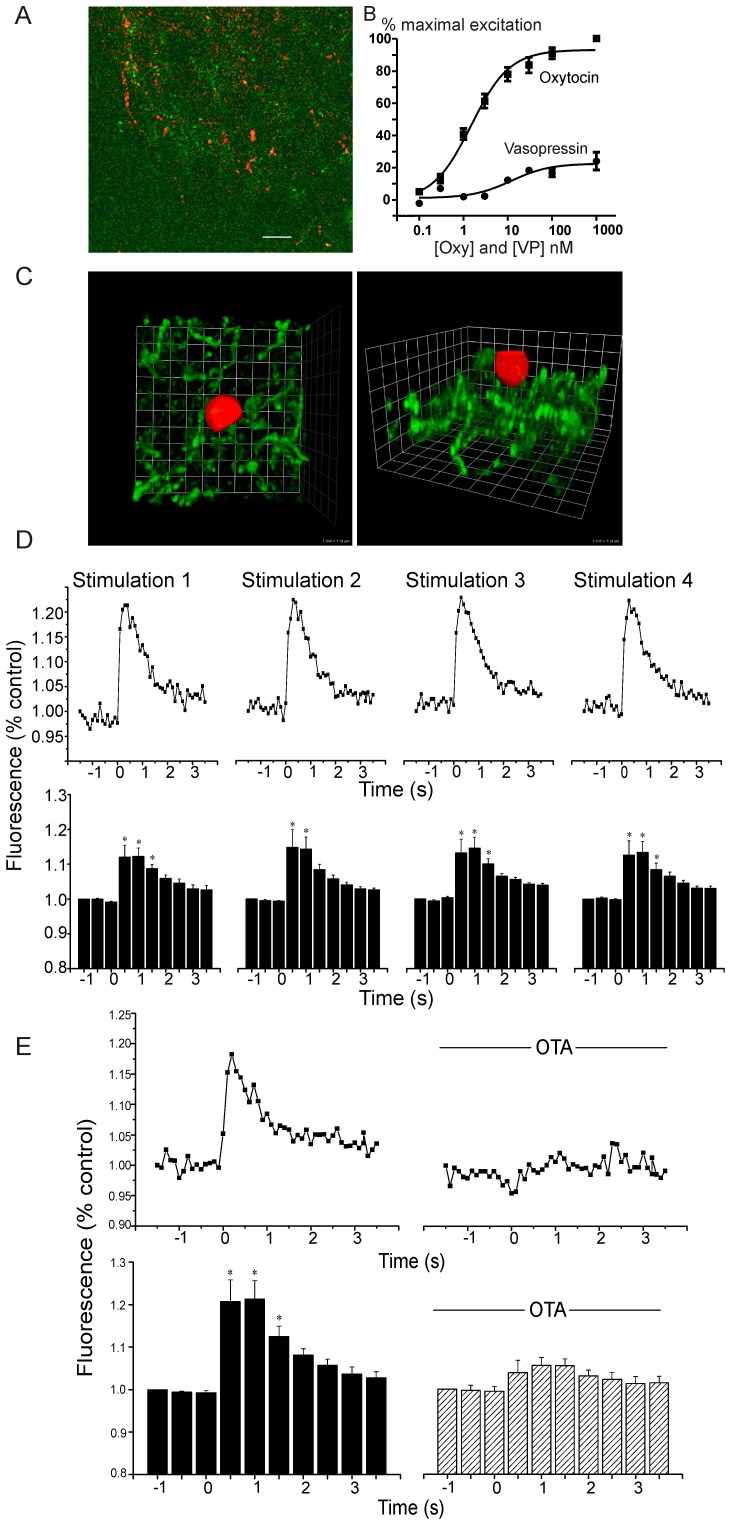
Imaging oxytocin and oxytocin receptor activation. (**A**) Co-localization of ChR2-EYFP and oxytocin in brainstem fibers within the DMNV is shown (scale bar represents 28 microns). 55.7+3.7% of ChR2-EYFP PVN fibers in the DMNV are positive for oxytocin. (**B**) Both the oxytocin and vasopressin dose-response relationships of sniffer CHO cells expressing an oxytocin receptor and a R-GECO Ca^2+^ indicator were characterized. These oxytocin receptor expressing CHO cells are considerably more sensitive and responsive to oxytocin than vasopressin with a half maximal response (EC-50) for oxytocin of 1.5 nM, and an EC-50 for vasopressin of 12.1 nM. Responses to oxytocin were considerably more robust than that for vasopressin; at the concentration (1 µM) at which oxytocin maximally activates these cells, vasopressin evoked a blunted response of only 24+5% of the oxytocin response. Sniffer CHO cells deposited on slices with ChR2 PVN fibers (green) in dorsal motor nucleus of the vagus (DMNV) (**C**) detect optogenetic oxytocin receptor activation in brainstem DMNV tissue in close apposition to PVN fibers, (3-D reconstruction top down view (left) and side view (right)). **D**, Repeated stimulations (5 min apart) of ChR2 axons in DMNV increased Ca^2+^. Representative traces of one sniffer CHO cell, top, and averages of repeated stimulations in 9 CHO cells, * p<0.0001, bottom. **E**, Oxytocin antagonist OTA blocks Ca^2+^ response, representative trace of one cell, top, and average control increase in 7 cells (; * p<0.0001), bottom.

As shown in figure 1A, 55.7+3.7% of ChR2-EYFP PVN fibers in the DMNV are positive for oxytocin. CHO cells that express both oxytocin receptors and the red fluorescent Ca^2+^ indicator R-GECO were utilized as sniffer cells for the synaptic release of oxytocin. As shown in the dose-response relationship in figure 1B, these oxytocin receptor expressing CHO cells are considerably more sensitive and responsive to oxytocin than vasopressin. The half maximal response (EC-50) of the CHO cells for oxytocin occurred at a concentration of 1.5 nM, whereas the EC-50 for vasopressin was 12.1 nM. In addition, responses to oxytocin were considerably more robust than that for vasopressin; at the concentration (1 µM) at which oxytocin maximally activates these cells, vasopressin evoked a blunted response of only 24+5% of the oxytocin response. Sniffer CHO cells were deposited onto the surface of brain stem slices, specifically on the dorsal vagal complex, containing both ChR2 expressing PVN fibers and CVNs in the DMNV (Figure 1C). Optogenetic stimulation of PVN fibers evoked large, reproducible, and transient increases in Ca^2+^ (average increase was 13.3±0.04% from baseline during first second; p = 0.0001; n = 9 cells) within the sniffer CHO cells (Figure 1D). The photostimulation-elicited increase in Ca^2+^ in the sniffer CHO cells upon PVN fiber activation was abolished by application of the oxytocin receptor antagonist OTA (control: 21.0±0.05%; OTA (10 µM): 4.6±0.02%; n = 7 cells; Figure 1E).

**Figure 2:**
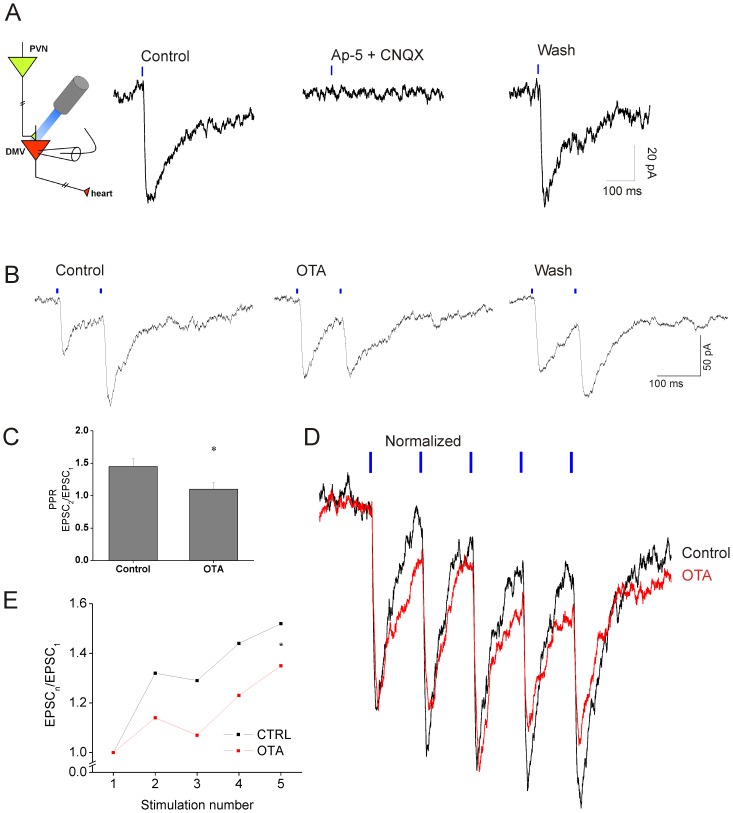
PVN monosynaptic glutamatergic projections to CVNs. **A**, Schematic of approach, left. Right, postsynaptic responses were completely blocked by antagonists for NMDA (AP-5; 50 µM) and AMPA/kainate receptors (CNQX; 50 µM). **B**, Paired-pulse facilitation (10 Hz) of glutamatergic neurotransmission from PVN to CVNs (first trace) abolished by oxytocin antagonist OTA (10 µM; middle trace) and partly restored upon wash out (right trace) **C**, Average data of OTA abolished paired-pulse facilitation (n = 7). * *p* = 0.021. **D**, Five consecutive stimulations (10 Hz;) with increasing amplitudes (black trace). OTA reduced the amplitude (red trace). Traces are normalized to amplitude of first response. **E**, Average data of the five stimulation paradigm (* *p* = 0.014)).

To determine the physiological role of the oxytocin receptor activation, if any, we photoactivated PVN fibers while recording post-synaptic synaptic currents in CVNs. Single pulse optogenetic stimulation (3 ms, 1 Hz) of PVN fibers in brain stem slices (Figure 2A) resulted in excitatory post-synaptic currents (EPSCs) in CVNs with a short latency (5.3±0.46 ms; n = 14). This single evoked response was reversibly abolished by the glutamate receptor antagonists AP-5 and CNQX (Figure 2A).

We used the paired-pulse response (PPR; calculated as amplitude of the response to the second stimulation/amplitude of the response to the first stimulation) paradigm to assess short-term plasticity of the PVN to CVN projection [17]. We observed paired-pulse facilitation of the response at stimulation frequencies of 0.5 Hz and 10 Hz (3 ms pulses), with average PPRs of 1.19±0.05 (n = 10) and 1.45±0.12 (n = 7), respectively. At both stimulation frequencies the second response was significantly larger (*p* = 0.004 and *p* = 0.049, respectively) than the first. To investigate whether oxytocin receptor activation influences this short-term synaptic plasticity of PVN to CVN projections, we examined the generation of paired pulse facilitation after application of the oxytocin receptor antagonist OTA (10 µM). OTA abolished paired-pulse facilitation, significantly reducing it from 1.45±0.12 to 1.10±0.10 (n = 7; *p* = 0.021; Figure 2B, C). In the presence of OTA the amplitude of the second response was not significantly different from the first (*p* = 0.787). OTA did not alter the amplitude of the response to the first stimulation (control: 57.5±8.41 pA; OTA: 63.4±12.27 pA; p = 0.453) or change the failure rate of the first or second response (failure rate control: first stimulation 9.0±5.0%, second stimulation 10.0±5.1%; failure rate OTA: first stimulation 7.2±4.2%, second stimulation 11.0±5.2%). Extending the stimulation protocol to five 3 ms pulses at a frequency of 10 Hz further increased the facilitation of glutamatergic neurotransmission from PVN fibers to CVNs (Figure 2D and E). The oxytocin receptor antagonist OTA significantly decreased this facilitation and reduced the amplitudes of sequential responses (when comparing the second through the fifth stimulation to the initial response, *p* = 0.014; n = 8, Figure 2D and E).

**Figure 3:**
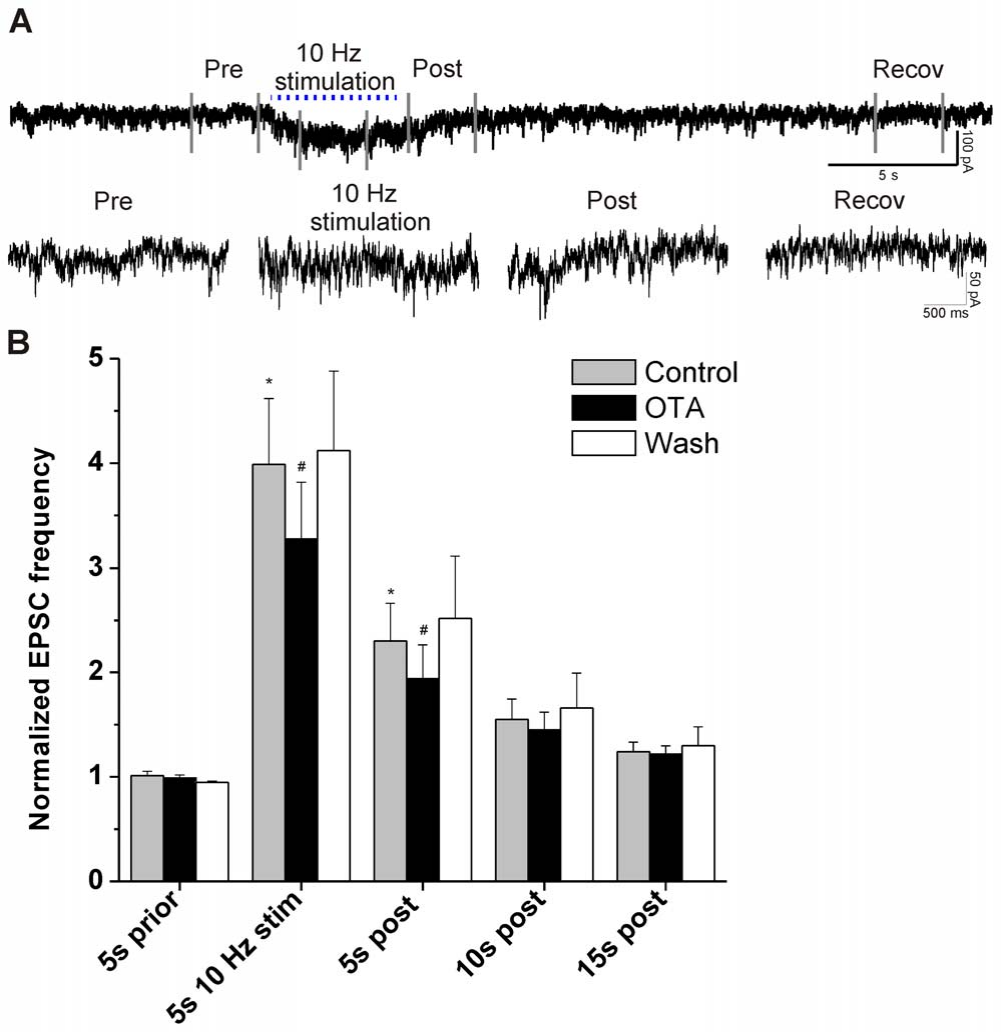
EPSCs during and after train stimulation. **A**, Upper trace: original recording with a 5 s train of stimuli (10 Hz,). Lower traces are details of Pre, 10 Hz Stimulation, Post and Recovery conditions of same recording. **B**, Group data of EPSCs. EPSC are increased during stimulation until at least 5 s post stimulation (* *p*<0.05 compared to Control 5 s prior). Oxytocin antagonist OTA reduces the EPSC frequency during and 5 s post stimulation (#, 5 s 10 Hz stim Control vs. OTA, *p* = 0.015; 5 s post Control vs OTA, *p* = 0.012).

To test if prolonged activation of PVN fibers would evoke long-lasting facilitation of glutamatergic synaptic events that continue beyond the period of stimulation we stimulated PVN terminals for 5 s and examined long-lasting alterations in the spontaneous EPSC event frequency. Prolonged stimulation of PVN terminals for 5 s (3 ms pulses, 10 Hz) caused sustained increase in the frequency of glutamatergic EPSCs both during and after stimulation (Figure 3). On average the EPSC frequency was increased 3.9 times during stimulation compared to prior (*p* = 0.002; n = 9) and remained significantly increased after cessation of PVN fiber stimulation until at least 5 seconds post stimulation (*p*<0.05). OTA significantly reduced, but did not abolish, the PVN fiber evoked facilitation of EPSC frequency during stimulation (18% decrease from 4.0±0.6 to 3.3±0.5; p = 0.015; n = 9; Fig. 3B), and this inhibition persisted for at least 5 s after stimulation (16% decrease from 2.3±0.40 to 1.9±0.3; *p* = 0.012; n = 9; Fig. 3B). This stimulation protocol did not reveal any postsynaptic fast ligand-gated responses other than those mediated by glutamate, since all optogenetically evoked post-synaptic currents were abolished in the presence of AP-5 and CNQX.

The observed oxytocin receptor mediated facilitation of the glutamatergic neurotransmission can be a unique property of PVN terminals that surround CVNs or it can be a general characteristic of glutamatergic neurotransmission to CVNs. To distinguish between these possibilities, a bipolar stimulation electrode was used to deliver paired-pulse activation of ubiquitous glutamatergic EPSCs in CVNs. The oxytocin receptor agonist TGOT (0.5 µM) did not alter the PPR with non-selective activation of glutamatergic inputs to CVNs (PPR control: 1.10±0.14; PPR TGOT 1.07±0.16; n = 7; *p* = 0.883) or the amplitude of the first response (control: 218.8±40.7 pA; TGOT 187.5±53.4 pA; n = 7; *p* = 0.462). These results indicate that the oxytocin receptor mediated facilitation of glutamatergic neurotransmission to cardioprotective CVNs was specific to the pathway originating from the PVN.

## Discussion

Our findings demonstrate that photostimulation of hypothalamic PVN fibers in brainstem parasympathetic nuclei activates oxytocin receptors, likely by co-release of oxytocin with glutamate, that facilitates excitation of cardioprotective CVNs. This neuropeptide-mediated synaptic plasticity is specific for this pathway and elicits increases in paired-pulse facilitation and long lasting spontaneous glutamatergic neurotransmission. As CVNs are intrinsically silent [18], facilitation of the major excitatory input to CVNs would likely play an important role in maintaining and increasing the firing of these neurons responsible for generating parasympathetic activity to the heart. Together, our data identifies a hypothalamic pathway capable of eliciting oxytocin receptormediated facilitation of CVNs, and suggests mechanisms that could facilitate or mimic activation of this pathway and receptors, which could be novel targets to mitigate the deleterious cardiovascular risks associated with stress and anxiety.

Although the PVN is well known as an important site for sympathetic cardiovascular regulation that include projections to pre-sympathetic neurons in the rostral ventrolateral medulla and pre-ganglionic neurons in the upper spinal cord [19]–[21], the hypothesis that different neurons in the PVN play a role in parasympathetic control of heart rate is contentious, in spite of evidence from neuroanatomical data that the PVN also sends dense axonal projections to parasympathetic nuclei in the brainstem [22]–[25]. In addition to the classic effects of oxytocin on uterine contraction and milk ejection, recent work indicates oxytocin is present in both males and females and has an important role in both behavior and cardiovascular homeostasis and parasympathetic cardiac activity, particularly during anxiety and stress [26]. In human volunteers in unstressed conditions intranasal administration of oxytocin significantly increases parasympathetic and decreases sympathetic cardiac control [27]. Oxytocin administration, in both men and women, increases trust, generosity, and willingness to cooperate [28]. In animal models of social stressors, oxytocin has been shown to be protective against behavioral and cardiac dysfunction. For example social isolation, which increases heart rate, diminishes HR variability and vagal regulation of the heart, was prevented with oxytocin administration [7], [8]. However whereas oxytocin reduces anxiety and the behavioral responses to stress, vasopressin increases anxiety and aggression, and enhances the responses to stressors [28], [29]. In the amygdala, a nucleus important in fear behavior and anxiety, vasopressin and oxytocin modulate the excitability of neurons in opposite ways via modulation of excitatory synaptic inputs to these neurons, providing a neurophysiologic mechanism for their opposing effects of these peptides on autonomic fear responses [30].

The results in this study identify one pathway in which oxytocin receptors are endogenously activated and act as an important neuromodulator of synaptic function. Optogenetic stimulation of PVN axons activates oxytocin receptors in sniffer oxytocin-sensitive CHO cells, and furthermore, oxytocin receptor activation is responsible for the facilitation of the EPSCs with paired-pulse and prolonged high frequency stimulations. This direct oxytocin receptor mediated modulation in the neurotransmission to CVNs from the hypothalamic PVN nucleus is selective for this synaptic connection, and does not occur upon non-selective activation of glutamatergic synapses to CVNs. While the sniffer CHO cells are ∼8 fold more sensitive and responsive to oxytocin than vasopressin, and both the responses in the sniffer CHO cells and paired pulse facilitation in cardiac vagal neurons are abolished with an oxytocin receptor antagonist, we cannot completely rule out the possibility that vasopressin, instead of or with oxytocin, is released from PVN fibers and activates oxytocin receptors.

The pre- and/or post-synaptic sites of action of oxytocin receptor activation in this pathway are still unknown. It has been shown that oxytocin facilitates AMPA-receptor subtype glutamatergic neurotransmission to lamina II neurons in the spinal cord [31]. The oxytocin receptor agonist TGOT increased the frequency of spontaneous and miniature EPSCs, indicating a presynaptic site of modulation by oxytocin in the spinal cord. Furthermore, oxytocin increases LTP in olfactory bulb and hippocampus and mediates glutamatergic plasticity in the medial prefrontal cortex [32]–[34]. In hippocampal slices oxytocin receptor activation contributes to local translation of protein kinase Mζ [33]. In another study oxytocin caused glutamatergic EPSC amplitude depression in the medial prefrontal cortex, likely mediated by the endocannabinoid retrograde signalling pathway [34]. This activity-dependent depression was turned into facilitation of glutamate EPSCs by oxytocin, possibly via postsynaptic activation of the oxytocin receptor. Similarly there are two possible sites of action of oxytocin that cause the facilitation effect that we observe. The first possibility is presynaptic activation of oxytocin receptors, which can be coupled to G_q/11_ class proteins and can raise presynaptic Ca^2+^ levels through activation of the phospholipase C/inositol triphosphate pathway. However, several other intracellular pathways and G-proteins can be activated upon oxytocin binding to its receptor [35]. A second possibility is that post-synaptic oxytocin receptor activation triggers the release of a retrograde signalling molecule from the post-synaptic site [36], a mechanism that has been described for some oxytocin synapses [34].

In conclusion our data provide direct evidence that oxytocin receptors are activated, most likely by endogenous release of oxytocin in the synaptic pathway from the PVN to CVNs, and the activation of oxytocin receptors mediates paired-pulse facilitation and prolonged high frequency activation within this glutamatergic neurotransmission to CVNs. Restoration of parasympathetic activity to the heart has recently emerged as a promising new therapeutic approach to inhibit the progression of cardiovascular diseases, including heart failure and risk of sudden cardiac death. Re-establishment of cardiac vagal activity prevents arrhythmias, decreases risk of sudden death, and protects against ischemia/reperfusion injury [37], [38]–[44], [45], [46].

This pathway offers a mechanistic foundation for the potential beneficial effects of endogenous oxytocin release in augmenting cardioprotective parasympathetic cardiac activity.
